# CD9 negatively regulates collective electrotaxis of the epidermal monolayer by controlling and coordinating the polarization of leader cells

**DOI:** 10.1093/burnst/tkad012

**Published:** 2023-07-24

**Authors:** Xiaoqiang Liu, Jinrui Yang, Meng Kong, Min Jiang, Luojia Liu, Jinghong Zhang, Ying Chen, Xu Chen, Ze Zhang, Chao Wu, Xupin Jiang, Jie Liu, Jiaping Zhang

**Affiliations:** Department of Plastic Surgery, State Key Laboratory of Trauma, Burns and Combined Injury, Southwest Hospital, Third Military Medical University (Army Medical University), 29 Gaotan Yan Street, Shapingba, 400038 Chongqing, China; Department of Plastic Surgery, State Key Laboratory of Trauma, Burns and Combined Injury, Southwest Hospital, Third Military Medical University (Army Medical University), 29 Gaotan Yan Street, Shapingba, 400038 Chongqing, China; Department of Plastic Surgery, State Key Laboratory of Trauma, Burns and Combined Injury, Southwest Hospital, Third Military Medical University (Army Medical University), 29 Gaotan Yan Street, Shapingba, 400038 Chongqing, China; Department of Plastic Surgery, State Key Laboratory of Trauma, Burns and Combined Injury, Southwest Hospital, Third Military Medical University (Army Medical University), 29 Gaotan Yan Street, Shapingba, 400038 Chongqing, China; Department of Plastic Surgery, State Key Laboratory of Trauma, Burns and Combined Injury, Southwest Hospital, Third Military Medical University (Army Medical University), 29 Gaotan Yan Street, Shapingba, 400038 Chongqing, China; Department of Plastic Surgery, State Key Laboratory of Trauma, Burns and Combined Injury, Southwest Hospital, Third Military Medical University (Army Medical University), 29 Gaotan Yan Street, Shapingba, 400038 Chongqing, China; Department of Plastic Surgery, State Key Laboratory of Trauma, Burns and Combined Injury, Southwest Hospital, Third Military Medical University (Army Medical University), 29 Gaotan Yan Street, Shapingba, 400038 Chongqing, China; Department of Plastic Surgery, State Key Laboratory of Trauma, Burns and Combined Injury, Southwest Hospital, Third Military Medical University (Army Medical University), 29 Gaotan Yan Street, Shapingba, 400038 Chongqing, China; Department of Plastic Surgery, State Key Laboratory of Trauma, Burns and Combined Injury, Southwest Hospital, Third Military Medical University (Army Medical University), 29 Gaotan Yan Street, Shapingba, 400038 Chongqing, China; Department of Plastic Surgery, State Key Laboratory of Trauma, Burns and Combined Injury, Southwest Hospital, Third Military Medical University (Army Medical University), 29 Gaotan Yan Street, Shapingba, 400038 Chongqing, China; Department of Plastic Surgery, State Key Laboratory of Trauma, Burns and Combined Injury, Southwest Hospital, Third Military Medical University (Army Medical University), 29 Gaotan Yan Street, Shapingba, 400038 Chongqing, China; Department of Plastic Surgery, State Key Laboratory of Trauma, Burns and Combined Injury, Southwest Hospital, Third Military Medical University (Army Medical University), 29 Gaotan Yan Street, Shapingba, 400038 Chongqing, China; Department of Plastic Surgery, State Key Laboratory of Trauma, Burns and Combined Injury, Southwest Hospital, Third Military Medical University (Army Medical University), 29 Gaotan Yan Street, Shapingba, 400038 Chongqing, China

**Keywords:** CD9, Electric fields, Collective migration, Wound healing, F-actin polarization, leader cells

## Abstract

**Background:**

Endogenous electric fields (EFs) play an essential role in guiding the coordinated collective migration of epidermal cells to the wound centre during wound healing. Although polarization of leadercells is essential for collective migration, the signal mechanisms responsible for the EF-induced polarization of leader cells under electrotactic collective migration remain unclear. This study aims to determine how the leader cells are polarized and coordinated during EF-guided collective migration of epidermal cell sheets.

**Methods:**

Collective migration of the human epidermal monolayer (human immortalized keratinocytes HaCaT) under EFs was observed via time-lapse microscopy. The involvement of tetraspanin-29 (CD9) in EF-induced fibrous actin (F-actin) polarization of leader cells as well as electrotactic migration of the epidermal monolayer was evaluated by genetic manipulation. Blocking, rescue and co-culture experiments were conducted to explore the downstream signalling of CD9.

**Results:**

EFs guided the coordinated collective migration of the epithelial monolayer to the anode, with dynamic formation of pseudopodia in leader cells at the front edge of the monolayer along the direction of migration. F-actin polarization, as expected, played an essential role in pseudopod formation in leader cells under EFs. By confocal microscopy, we found that CD9 was colocalized with F-actin on the cell surface and was particularly downregulated in leader cells by EFs. Interestingly, genetic overexpression of CD9 abolished EF-induced F-actin polarization in leader cells as well as collective migration in the epidermal monolayer. Mechanistically, CD9 determined the polarization of F-actin in leader cells by downregulating a disintegrin and metalloprotease 17/heparin-binding epidermal growth factor-like growth factor/epidermal growth factor receptor (ADAM17/HB-EGF/EGFR) signalling. The abolished polarization of leader cells due to CD9 overexpression could be restored in a co-culture monolayer where normal cells and CD9-overexpressing cells were mixed; however, this restoration was eliminated again by the addition of the HB-EGF-neutralizing antibody.

**Conclusion:**

CD9 functions as a key regulator in the EF-guided collective migration of the epidermal monolayer by controlling and coordinating the polarization of leader cells through ADAM17/HB-EGF/EGFR signalling.

Highlights

- This is the first study to reveal that EFs guide collective migration of the epidermal monolayer by downregulating CD9 in leader cells.
- CD9 controls the EF-induced polarization of F-actin in leader cells by negatively regulating the sheddase of HB-EGF.
- CD9-mediated HB-EGF secretion might coordinate the EF-induced polarization of leader cells through paracrine effects.

## Background

An essential feature of a healed wound is the restoration of an intact epidermal barrier through wound epithelialization, also known as re-epithelialization. The directed migration of keratinocytes is critical to wound re-epithelialization, and defects in this function are associated with the clinical phenotype of chronic nonhealing wounds [1,2]. Directional migration of epidermal cells towards the wound centre is a response to various chemical and physical factors in the wound microenvironment [3]. Endogenous electric fields (EFs) are known to play an important role in wound healing, mainly through their effects on cell migration [4,5]. In wounds of epidermal tissue, endogenous EFs are instantaneously generated after injury due to the disruption of transepithelial potentials, causing the wound centre to become more negatively charged than the surrounding area. Electric signals can override other cues to direct the migration of epithelial cells, a phenomenon called cell electrotaxis or galvanotaxis [6–8].

The molecular mechanisms underlying the EF-induced directional migration of isolated cells have been widely studied *in vitro*. However, epithelial cells migrate collectively as a coherent sheet to heal wounds *in vivo*. Collective migration is fundamentally different from the migration of isolated cells. Interestingly, it has been found that the epithelial sheets respond to an EF significantly better—more directionally and efficiently—than cells in isolation, indicating better electrotaxis in the epithelial sheets than in the isolated cells [9–11]. However, it is still largely unknown how EFs guide the collective migration of cell sheets and how this effect is more efficient than that of isolated cells.

Collective migration involves coordination between two functionally distinct cell populations: leader cells and follower cells. The leader cells localize at the front of a moving group, where they receive guidance signals and migrate through instructing actin-based structures such as pseudopodia in the direction of cell movement [12]. The follower cells, however, migrate following the leader cells by sensing the guiding signals from the leader cells [13]. The formation of pseudopodia (lamellipodia and filopodia in keratinocytes) in leader cells is driven by fibrous actin (F-actin) polymerization and polarization, a process tightly regulated by signalling complexes [14]. Tetraspanin-29 (CD9), a member of the tetraspanin superfamily, has been shown to colocalize with cytoskeletal F-actin in the filopodia on the cell surface, where it regulates the arrangement of the actin cytoskeleton [15,16]. However, CD9 lacks receptor function compared to numerous other membrane surface proteins. It has been suggested that CD9 might engage in the organization of polyprotein complexes on the surface via combination with other molecules, thus mediating a variety of physiological and cellular processes [17–19]. In our previous study, we determined that CD9 and a disintegrin and metalloprotease 17 (ADAM17) colocalized on the surface of keratinocytes, and the sheddase activity of ADAM17 was activated by CD9 downregulation [20]. ADAM17, a known ectodomain sheddase of epidermal growth factor receptor (EGFR) ligands such as heparin-binding epidermal growth factor-like growth factor (HB-EGF), has been reported to enhance actin cytoskeletal remodelling at the tip of the lamellipodium in hepatocellular carcinoma (HCC) cells [21,22]. We have also shown the important role of ADAM17-driven HB-EGF/EGFR signalling in the EF-guided collective migration of epidermal sheets [23]. Nevertheless, the mechanisms by which the polarization of leader cells is controlled in EF-guided collective migration of the epidermal monolayer remain to be elucidated. Intercellular communication is crucial for the efficiency of collective migration [2,24,25]. Paracrine signalling is a main form of intercellular communication, by which cells respond to the factors produced from nearby cells [26,27]. It has been shown that HB-EGF plays a variety of roles in cell proliferation, migration and inflammation through paracrine action [28,29]. Whether such a paracrine mechanism between leader cells coordinates their polarization and behaviour under EFs also needs to be investigated.

In this study, we found that CD9 colocalized with F-actin and was particularly downregulated in the leader cells of EF-guided migrating epithelial monolayers. The downregulation of CD9 facilitated F-actin polarization in leader cells by activating ADAM17/HB-EGF/EGFR signalling, thus supporting the directional migration of the epithelial monolayer under EFs. This CD9-mediated signalling coordinated the polarization of leader cells through HB-EGF paracrine signalling, providing new insights into how collective migration is initiated and coordinated under EFs.

## Methods

### Cell culture

Human immortalized keratinocytes HaCaT cells were purchased from the Cell Bank of the Chinese Academy of Sciences and were cultured in RPMI 1640 medium containing 100 μg/ml streptomycin, 100 U/ml penicillin and 10% fetal bovine serum. Cells were incubated at 37°C, 95% humidity and 5% CO_2_.

### EF stimulation and time-lapse image recording

To observe the migration of HaCaT cells, an experimental chamber previously developed by our group was used [30,31]. To stimulate the cells by EFs, we generated EFs using two carbon fibre electrodes immersed in Steinberg solution (0.7 mM KCl, 0.8 mM MgSO_4_, 60 mM NaCl, 0.3 mM CaNO_3_-4H_2_O and 1.4 mM Tris base, pH 7.4) and connected the electrodes to the medium by two salt bridges (Steinberg’s solution containing 2% agar). The cells were placed in an EF, adjusted to the required EF intensity, and stimulated for 6h. Migration of the cell monolayer was monitored using a Zeiss time-lapse imaging system (Carl Zeiss Meditec, Jena, Germany). Images were analysed by ImageJ.

### Quantitative analysis of cell-migration directivity and velocity

The movement of HaCaT cells was analysed by ImageJ software. The central position of the cells was tracked and the starting point was set as the origin. The directivity of the cells was quantified by Cosθ, which represents the angle between the field vector and the line connecting the starting point to the end point of the cells. Cosθ ranges from −1 to +1, with 1 indicating that the cells migrate towards the cathode, −1 indicating that the cells migrate towards the anode, and near 0 indicating random migration without direction. Displacement velocity (Td/t) is the absolute X-value of the cell endpoint position divided by time, indicating the migration velocity of cells along the EF vector. Trajectory velocity (Tt/t) is the total length of the trajectory of cell migration divided by time, indicating the migration velocity of cells.

### CD9-overexpressing recombinant adenovirus transfection

Ad-CD9-GFP and the CD9 mimic vector Ad-GFP were obtained from Shanghai Gene Chemistry. Briefly, HaCaT cells were inoculated in 6-well plates at a cell density of 30–50%, and HaCaT cells were infected with CD9-overexpressing recombinant adenovirus (Ad-CD9-GFP) and CD9 mimic vector (Ad-GFP) for 48 h. The cells were divided into the control group (both Ad-CD9-GFP and Ad-GFP were not transfected), vector group (transfected with Ad-GFP), and Ad-CD9 group (transfected with Ad-CD9-GFP) according to the treatment conditions. After 48 h of infection, it was confirmed that >90% of HaCaT cells were infected by observing  green fluorescent protein (GFP) expression by fluorescence microscopy (Figure S2a). Western blots also verified effective overexpression of the CD9-GFP fusion protein (Figure S2b, c). The molecular weight of CD9-GFP was ~55 kDa.

### Fluorescence labelling and co-culture of adherent cells

HaCaT cells were labelled with a cell plasma membrane staining kit with DiI (red fluorescence) and a cell plasma membrane staining kit with DiO (green fluorescence) according to the manufacturer’s protocol (Shanghai Beyotime Biotechnology). Briefly, the adherent cells in the 6-well plate were washed 2–3 times with sterile phosphate buffer solution (PBS). Staining working solution (1 ml) was added, gently shaken to cover all cells, and incubated at 37°C in the dark for 15–20 min. HaCaT cells labelled with DiI and DiO were then co-seeded in the EF chamber, cultured into monolayer and placed in a Zeiss time-lapse imaging system to observe cell migration.

### ADAM17 activity determination

ADAM17 activity was determined using an enzyme-linked immunosorbnent assay (ELISA) kit for ADAM17 (Yun clone Technology) according to the manufacturer’s protocol. Briefly, each group of cell extracts was incubated in anti-human ADAM17 antibody-coated 96-well plates for 1 h at room temperature, washed well, patted dry and then incubated with fluorescently labelled substrate at 37°C. The relative optical density values were detected at an emission wavelength of 450 nm using a multifunctional enzyme standardizer, and these data were normalized relative to the control group.

### Western blot analysis

HaCaT cell monolayers were washed with precooled PBS, collected in 100–200 μl of protein lysis solution, lysed on ice for 20–30 min and sonicated for 4 s. The supernatant was collected by centrifugation. The protein concentration was determined by a bicinchoninic acid assay (BCA) protein analysis kit (Sigma, USA). Equal amounts of proteins were separated by 10% sodium dodecyl sulfate polyacrylamide gel electrophoresis (SDS-PAGE) and transferred to polyvinylidene difluoride membranes, which were blocked with 5% skimmed milk for 2 h at room temperature, incubated with primary antibody at 4°C overnight and incubated with secondary antibody for 1 h at room temperature for detection of the blots. Primary antibodies were as follows: CD9 (1 : 500, Santa, UK), EGFR (1 : 1000, Abcam, UK), phospho-EGFR (1 : 1000, Abcam, UK) and β-actin (1 : 1000, CST, USA). Secondary antibodies were as follows: anti-mouse IgG (1 : 2500, CST, USA) and anti-rabbit IgG (1 : 2500, CST, USA).

### Pharmacological reagent treatment

HaCaT cells were cultured into monolayers and treated with a final concentration of 40 μM ADAM17 inhibitor TNF protease inhibitor 2 (TAPI-2; MCE, USA), 20 μM EGFR inhibitor AG1478 (MCE, USA), 5 μg/ml of the F-actin polymerization inhibitor cytochalasin B (CB; MCE, USA) or 100 ng/ml of recombinant human heparin-binding EGF (MCE, USA). Before performing the HB-EGF antibody neutralization experiment, HB-EGF in the supernatant was removed.

### Immunofluorescence assay

Treated HaCaT cell monolayers were washed with precooled PBS at 4°C, then incubated in 4% paraformaldehyde at room temperature for 20–30 min, incubated with 10% goat serum at room temperature for 1 h and incubated overnight at 4°C with primary antibody CD9 (1 : 100, Santa, UK). After staining for 1 h in the dark with a fluorescent secondary antibody (1 : 500, Abcam, UK), rhodamine-labelled phalloidin (1 : 1000, Proteintech, USA) and 4',6-diamidino-2-phenylindole (DAPI, 1 : 1000, Sigma, USA), the cells were observed under a laser confocal microscope (Olympus) and photographed.

### Statistical analysis

Data were obtained from at least three independent replicate experiments and statistically analysed using SPSS 24.0 statistical software. Measurement data are expressed as mean ± standard deviation (SD) or median (interquartile range); Student’s t-test or the Mann–Whitney U test was used for comparisons of two independent samples, one-way ANOVA (Analysis of variance) or the Kruskal–Wallis H test was used for comparison of multiple independent samples. The least-significant difference t test or Nemenyi test was used for *post hoc* multiple comparison after one-way ANOVA or Kruskal–Wallis H test. Values of *p* < 0.05 were considered statistically significant and statistical plots were drawn using GraphPad Prism V.5.

## Results

### EFs guided collective migration of the epidermal monolayer in a voltage-dependent manner

**Figure 1:**
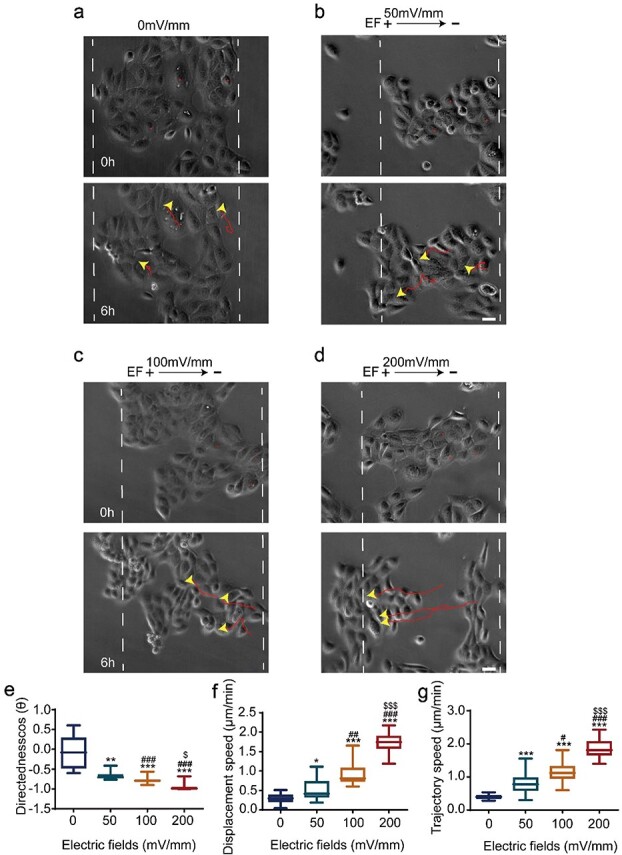
EFs induced collective migration of HaCaT monolayer to the anode directionally. (**a**–**d**) Representative images of the collective migration of HaCaT monolayer under different EF strengths (0, 50, 100 and 200 mV/mm); yellow arrowheads and red curves indicate the cell-migration trajectories. Analysis of (**e**) migration directionality, (**f**) displacement velocity and (**g**) trajectory velocity. ^*^, ^*^^*^, ^*^^*^^*^p < 0.05, 0.01 and 0.001, respectively, *vs* the 0 mV/mm group; #, ##, ###*p* < 0.05, 0.01 and 0.001, respectively, *vs* the 50 mV/mm group; ^$, $$, $$$^*p* < 0.05, 0.01 and 0.001, respectively, *vs* the 100 mV/mm group. Scale bars 10 μm (see supplementary movie S1). *EFs* electrical fields, *HaCaT* human immortalized keratinocytes

To assess the electrotactic behaviour of the epidermal monolayer, we first visualized migration of the HaCaT monolayer with or without EF application via time-lapse microscopy. In the no EF-treated monolayer (0 mV/mm), the migration of the HaCaT monolayer was random, with no significant directional migration (Figure 1a, and Figure S1 and movie S1, see online supplementary material). In an EF of 50–200 mV/mm, the monolayer migrated in a collective manner towards the anode directionally (Figure 1b–d and movie S1). We then ascertained whether the collective electrotactic behaviour was voltage dependent. In monolayers exposed to EFs with strengths of 50, 100 and 200 mV/mm, the directionality (cos(Ɵ)), displacement velocity (Td/t) and trajectory velocity (Tt/t) of the HaCaT monolayer increased gradually, reaching maximums at 200 mV/mm with values of −0.98 (0.04), 1.74 (0.31) μm/min and 1.81 (0.41) μm/min, respectively (Figure 1e–g). These results indicate that EFs drive the epidermal monolayer to migrate towards the anode collectively in a field strength-dependent manner. An EF of 200 mV/mm was thus selected as the optimal strength for the subsequent experiments.

### F-actin polarization is required for the formation of leader cells at the front edge of the migrating monolayer under an EF

**Figure 2:**
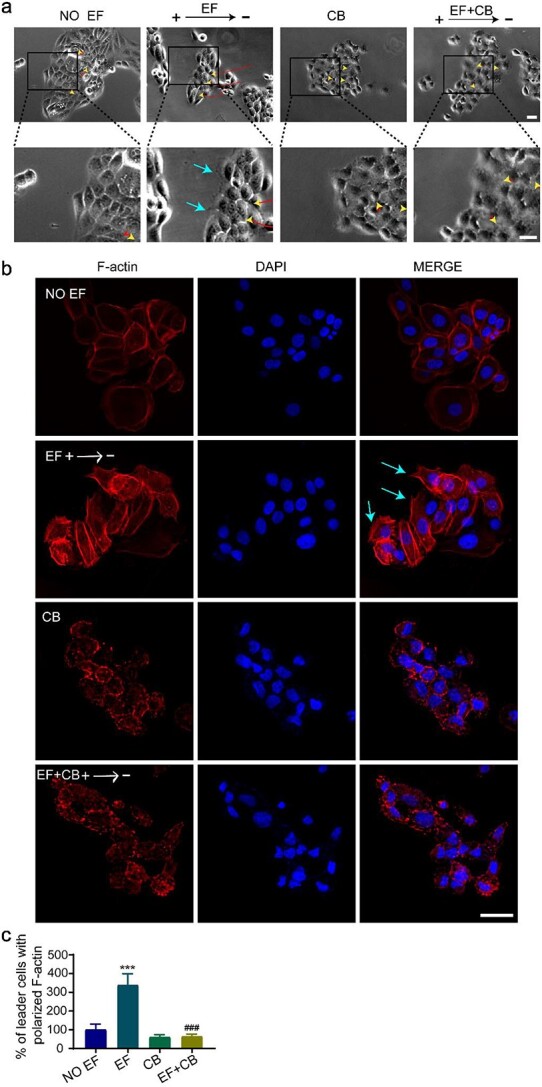
F-actin polarization was required for leader cells formation at the front edge of migrating monolayer under EFs. (**a**) Representative images of the collective migration of HaCaT monolayer; yellow arrowheads and red curves indicate the cell-migration trajectories, cyan arrows indicate pseudopodia. (**b**) Immunofluorescence staining of F-actin in HaCaT monolayer; cyan arrows indicate F-actin polarization. (**c**) Statistical analysis indicating the F-actin distribution of HaCaT monolayer. ^*^^*^^*^p< 0.001 *vs* the no EF group; ^###^*p* < 0.001 *vs* the EF group. Scale bars 10 μm (see supplementary movie S2). *F-actin* fibrous actin, *EFs* electrical fields, *HaCaT* human immortalized keratinocytes

The formation of pseudopodia, characterized by F-actin polarization along the direction of migration at the front edge of the cell monolayer, is essential for the onset of directed collective migration [12,32]. Cells with polarized F-actin and pseudopodia are called leader cells. Time-lapse recording showed that the anodic side cells in the EF-treated HaCaT monolayer formed polarized pseudopodia along the direction of migration compared to the no-EF control. By immunofluorescence staining, significant F-actin polarization was found in the pseudopodia at the anodic side cells in the EF-treated HaCaT monolayer [Figure 2a, b, and movie S2 (see online supplementary material), cyan arrows indicate pseudopodia]. Polarized F-actin was increased 3.4-fold in the EF-treated monolayer compared to the no-EF control (Figure 2c). These results suggested a typical formation of leader cells induced by EFs in the epidermal monolayer. Inhibition of F-actin polarization by CB, a specific F-actin polymerization inhibitor, abolished the EF-induced pseudopodia in leader cells as well as the EF-induced collective migration of monolayers (Figure 2a, b and movie S2), suggesting that F-actin polarization is a prerequisite for the formation of leader cells in the collective migration of epidermal monolayers under EFs.

### CD9 colocalizes with F-actin and is particularly downregulated in leader cells by EFs

**Figure 3:**
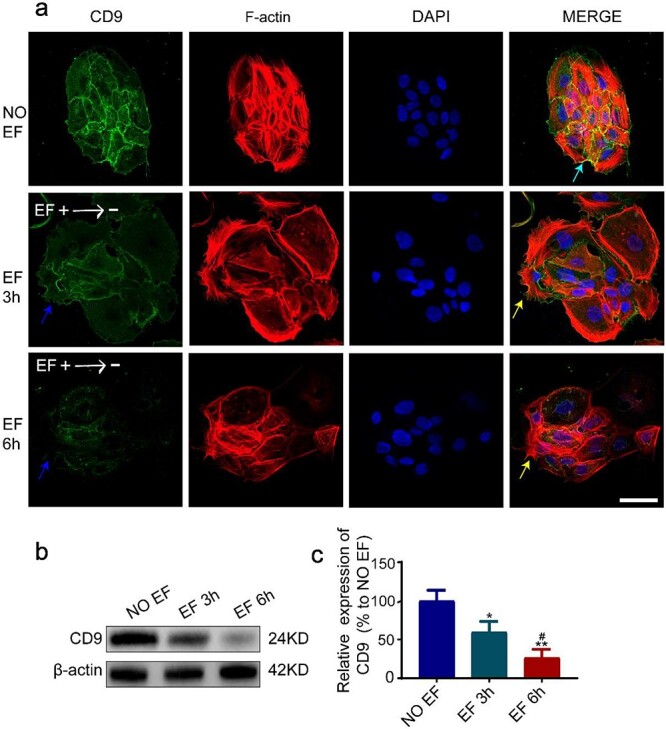
CD9 co-localized with F-actin and was down-regulated in leader cells by EFs. (**a**) Immunofluorescence staining of CD9 and F-actin in HaCaT monolayer; cyan arrows indicate colocalization of CD9 and F-actin, yellow arrows indicate polarized F-actin and pseudopodia. (**b**) The expression of CD9 in HaCaT monolayer was determined by western blot. (**c**) The results were quantified by relative intensity. ^*^, ^*^^*^p< 0.05 and 0.01, respectively, *vs* the no EF group; #*p* < 0.05 *vs* the EF 3 h group. Scale bars 10 μm. *CD9* Tetraspanin-29, *F-actin* fibrous actin, *EFs* electrical fields, *HaCaT* human immortalized keratinocytes

Previous studies have demonstrated that CD9 colocalizes with cytoskeletal F-actin in the filopodia of the cell surface and regulates the arrangement of the actin cytoskeleton [15]. We have shown that CD9 downregulation contributes to the migration of epidermal cells [20]. We hypothesized that EF-induced polarization of F-actin in the leader cells is associated with CD9. To test this hypothesis, we co-labelled CD9 and F-actin by immunofluorescence staining. As shown in Figure 3a, CD9 and F-actin showed obvious colocalization at the membrane of epidermal cells in a normal cultured monolayer (no-EF control, shown by cyan arrows). After EF treatment, CD9 staining was significantly attenuated as a whole when compared to the no-EF control (Figure 3a). This result was also confirmed by western blotting, which showed that the protein level of CD9 was significantly decreased in a time-dependent manner in EF-treated monolayers (Figure 3b, c). Particularly, the leader cells, which formed distinct polarized F-actin and pseudopodia along the direction of motion (shown by yellow arrows), showed lower CD9 staining (shown by blue arrows) than the following cells (Figure 3a). These results indicate that EFs induce a low level of CD9 in the epidermal monolayer, which may be involved in the formation of cellular F-actin and pseudopodia in the leader cells and therefore benefit the EF-guided collective migration of the monolayer.

### Upregulation of CD9 inhibits F-actin polarization of leader cells and collective migration of the epidermal monolayer under EFs

**Figure 4:**
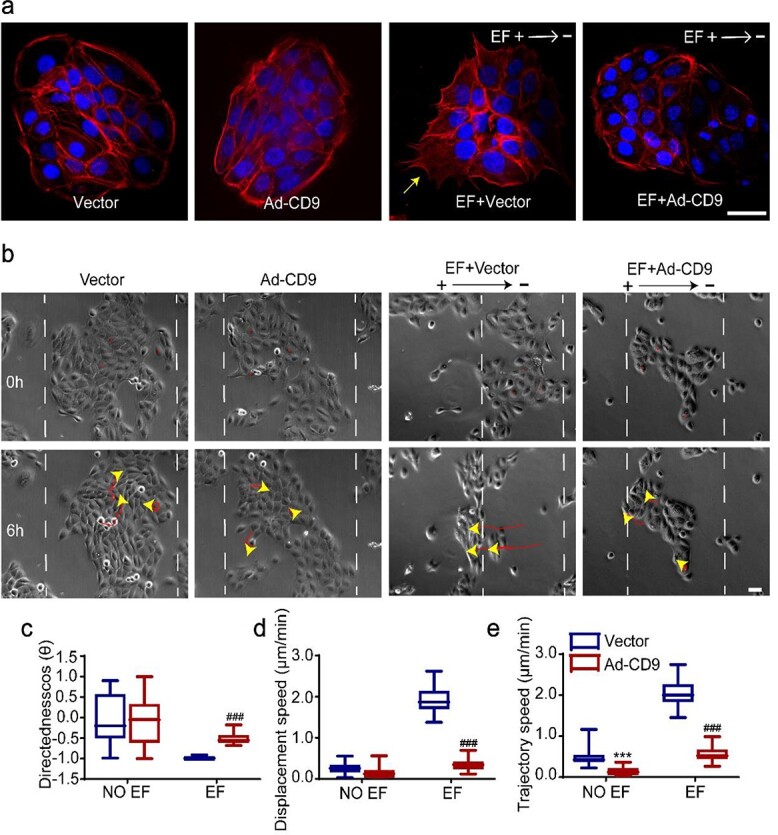
Up-regulation of CD9 inhibited F-actin polarization of leader cells and collective migration of epidermal monolayer under EFs. (**a**) Immunofluorescence staining of F-actin in HaCaT monolayer; yellow arrows indicate polarized F-actin and pseudopodia. (**b**) Representative images of the collective migration of HaCaT monolayer; yellow arrowheads and red curves indicate the cell-migration trajectories. Analysis of (**c**) migration directionality values of HaCaT monolayer, (**d**) displacement velocity of HaCaT monolayer and (**e**) trajectory velocity of HaCaT monolayer. ^*^^*^^*^p < 0.001 *vs* the vector group; ###*p* < 0.001 *vs* the EF + vector group. Scale bars 10 μm (see supplementary movie S3). *CD9* Tetraspanin-29, *F-actin* fibrous actin, *EFs* electrical fields, *HaCaT* human immortalized keratinocytes, *Ad-CD9* adenovirus vector for overexpressing CD9

To determine whether the EF-induced F-actin polarization was regulated by CD9, a recombinant adenoviral vector overexpressing CD9 (Ad-CD9) was constructed and used to infect the HaCaT monolayer before EF treatment (Figure S2, see online supplementary material). As shown in Figure 4a, EF-induced F-actin polarization and pseudopod formation in the leader cells of the monolayer were mostly inhibited by CD9 overexpression (the yellow arrow indicates F-actin polarization). The directed collective migration of the monolayer was largely abolished by CD9 overexpression (Figure 4b, and Figure S3 and movie S3, see online supplementary material). The directionality (cosƟ) decreased from −0.99 (0.01) to −0.55 (0.17) (Figure 4c), the displacement velocity (Td/t) decreased from 1.87 (0.42) to 0.34 (0.16) μm/min (Figure 4d) and the trajectory velocity (Tt/t) decreased from 2.00 (0.41) to 0.52 (0.21) μm/min (Figure 4e). Our findings suggest that CD9 negatively regulates collective electrotaxis by the epidermal monolayer by inhibiting F-actin polarization and pseudopod formation in the leader cells.

### CD9 controls the F-actin polarization of leader cells by negatively modulating the ADAM17/HB-EGF/EGFR axis

**Figure 5:**
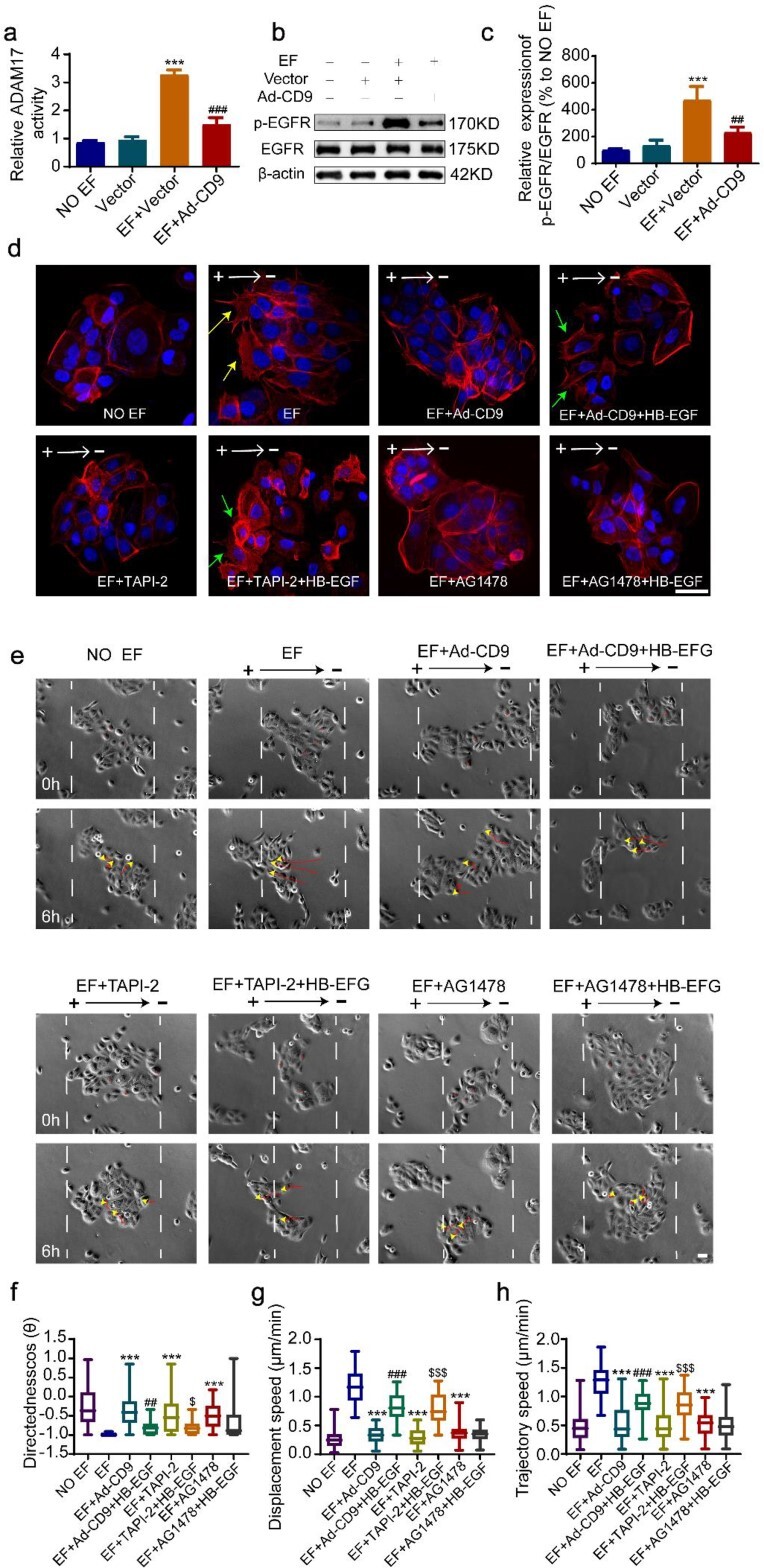
CD9 controlled F-actin polarization of leader cells by negatively modulating the ADAM17/HB-EGF/EGFR axis. (**a**) ADAM17 activity of HaCaT monolayer under EFs. (**b**) The expression of p-EGFR was determined by western blot. (**c**) Results were quantified by relative intensity. ^*^^*^^*^p < 0.001 *vs* the vector group; ##, ###*p* < 0.01 and 0.001, respectively, *vs* the EF + vector group. (**d**) Immunofluorescence staining of F-actin in HaCaT monolayer; yellow and green arrows indicate polarized F-actin and pseudopodia. (**e**) Representative images of the collective migration of HaCaT monolayer under EFs; yellow arrowheads and red curves indicate the cell-migration trajectories. Analysis of (**f**) the migration directionality of HaCaT monolayer, (**g**) displacement velocity of HaCaT monolayer and (**h**) trajectory velocity of HaCaT monolayer. ^*^^*^^*^p< 0.001 *vs* the EF group; ##, ###*p* < 0.01 and 0.001, respectively, *vs* the EF + Ad-CD9 group; ^$, $$$^*p* < 0.05 and 0.001, respectively, *vs* the EF + TAPI-2 group. Scale bars 10 μm (see supplementary movie S3). *CD9* Tetraspanin-29, *F-actin* fibrous actin, *ADAM17* a disintegrin and metalloprotease domain 17, *HB-EGF* heparin-binding EGF-like growth factor, *EGFR* epidermal growth factor receptor, *p-EGFR* phospho epidermal growth factor receptor, *TAPI-2* TNF protease inhibitor 2, *AG1478* EGFR tyrosine kinase inhibitors, *EFs* electrical fields, *HaCaT* human immortalized keratinocytes, *Ad-CD9* adenovirus vector for overexpressing CD9

We have previously demonstrated that CD9 negatively modulates the sheddase activity of ADAM17 to promote the shedding of HB-EGF and activation of EGFR in keratinocytes [20]. We hypothesized that CD9 controls EF-induced F-actin polarization in leader cells by negatively modulating the ADAM17/HB-EGF/EGFR axis. As shown in Figure 5a–c, EF exposure resulted in a significant increase in the activation of ADAM17 and EGFR in the epidermal monolayer, which was largely suppressed by CD9 overexpression, confirming a role for CD9 in the regulation of ADAM17 and EGFR under an EF. Immunofluorescence staining showed that F-actin was rapidly redistributed in the extended pseudopodia at the anodal side of the leader cells after exposure to the EF (Figure 5d, yellow arrows). Overexpression of CD9 and treatment with TAPI-2 (a specific ADAM17 inhibitor) or AG1478 (an EGFR tyrosine kinase inhibitor) completely disrupted the polarization of F-actin (Figure 5d). Interestingly, the disrupted polarization of F-actin by CD9 overexpression or TAPI-2 treatment, but not by AG1478 treatment, was restored by the addition of recombinant HB-EGF (Figure 5d, green arrows), suggesting that CD9-mediated ADAM17/HB-EGF/EGFR signalling is responsible for F-actin polarization in leader cells under EFs. Meanwhile, time-lapse recording showed that the collective electrotactic response of the monolayer was significantly decreased by CD9 overexpression and TAPI-2 or AG1478 pretreatment. The addition of recombinant HB-EGF, however, rescued the electrotaxis suppressed by CD9 overexpression or TAPI-2 pre-treatment (Figure 5e–h, and supplementary Figure S4 and movie S3, see online supplementary material). Taken together, these results clearly demonstrate that EFs activate the ADAM17/HB-EGF/EGFR axis by downregulating CD9 to induce F-actin polarization, leading to the induction of leader cells and the consequent directional collective migration of monolayers.

### The paracrine effect of HB-EGF released through CD9-mediated signalling coordinates the polarization of leader cells

**Figure 6:**
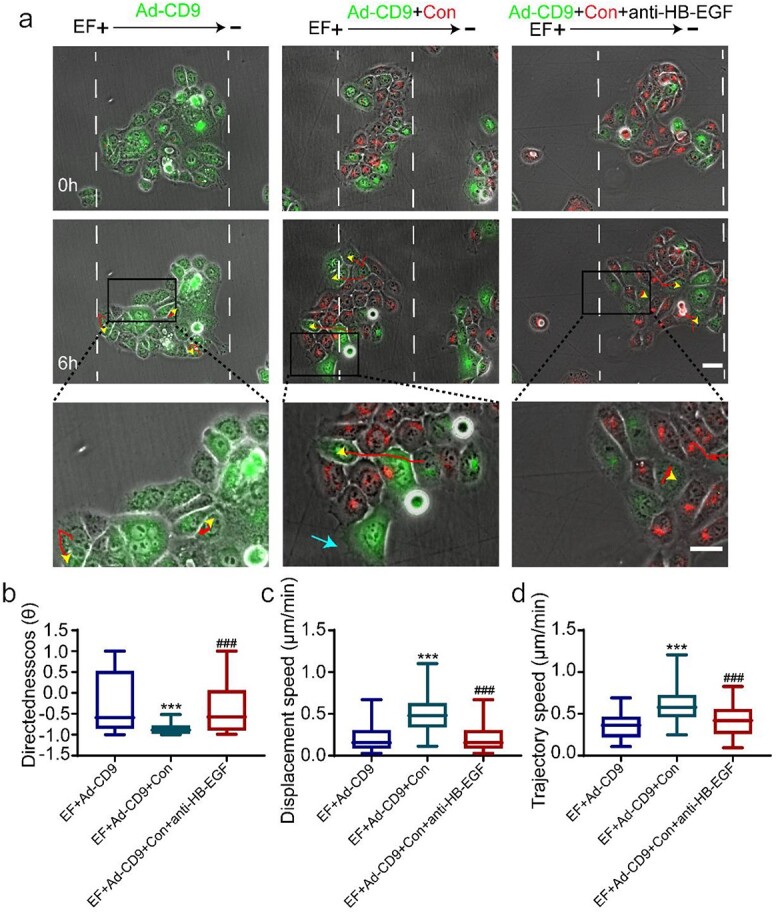
Paracrine effect of HB-EGF released through CD9-mediated signalling coordinated the polarization of leader cells. (**a**) Representative images of the collective migration of monolayer under EFs; yellow arrowheads and red curves indicate the cell-migration trajectories, cyan arrows indicate pseudopodia. Analysis of (**b**) migration directionality values of monolayer, (**c**) displacement velocity of monolayer and (**d**) trajectory velocity of monolayer. ^*^^*^^*^p < 0.001 *vs* the EF + Ad-cd9 group; ###*p* < 0.001 *vs* the EF + Ad-CD9 + Con group. Scale bars 10 μm (see supplementary movies S4). *CD9* tetraspanin-29, *HB-EGF* heparin-binding EGF-like growth factor, *anti-HB-EGF* heparin-binding EGF-like growth factor antibody, *Con* Control, *HaCaT* Human immortalized keratinocytes, *EF* electrical field, *Ad-CD9* adenovirus vector for overexpressing CD9

The epithelial monolayer is known to exhibit greater sensitivity and more effective electrotaxis than isolated cells, suggesting that there must be some communication mechanism between cells to coordinate the electrotaxis behaviour of collective cells. Paracrine signalling is an important form of intercellular communication by which cells can elicit responses to the factors produced from nearby cells [9,10,26,27]. In this study, we have demonstrated that EFs promote HB-EGF secretion by downregulating CD9 to induce F-actin polarization in leader cells. Therefore, we hypothesized that there is a mechanism undertaken by the leader cells to coordinate their polarization through a paracrine effect of HB-EGF. To explore this, we labelled CD9-overexpressing cells with DiO (green fluorescence, Ad-CD9) and normal HaCaT cells with DiI (red fluorescence, Con), and co-cultured the two cell lines to form a mixed-cell monolayer. The movement behaviour of cells in the mixed-cell monolayer under EFs was observed by time-lapse microscopy. As shown in Figure 6a, the CD9-overexpressing monolayer showed no pseudopod formation in cells at the anodal side of the EF, indicating failed induction of leader cells. As a result, no directional migration in the CD9-overexpressing monolayer was observed under the EF. However, the pseudopodia in CD9-overexpressing cells (shown by cyan arrows) at the anodal side of EFs were restored in the co-culture monolayer, indicating that the CD9-overexpressing cells regained the ability to become leader cells (Figure 6a, and supplementary Figure S5 and movie S4, see online supplementary material). Compared to the cells in the Ad-CD9 monolayer, the Ad-CD9 cells in the co-culture system increased their directionality (cosƟ) from −0.58 (1.46) to −0.89 (0.17) (Figure 6b), their displacement velocity (Td/t) from 0.16 (0.21) to 0.48 (0.28) μm/min (Figure 6c) and their trajectory velocity (Tt/t) from 0.35 (0.23) to 0.58 (0.25) μm/min (Figure 6d). The electrostatic response of the mixed-cell monolayer in migration directedness was comparable to that of the normal HaCaT monolayer (Figure 6b vs. Figure 1e, and supplementary Figure S5 and movie S4). The results indicated that the normal HaCaT cells in the mixed-cell monolayer produced a highly coordinated effect, enabling CD9-overexpressing cells in the mixed-cell monolayer to regain electrotaxis. Interestingly, the ability to become leader cells and the restoration of electrotaxis in the CD9-overexpressing cells in the mixed monolayer could be completely inhibited by addition of the HB-EGF neutralizing antibody (Figure 6a, b, and supplementary Figure S5 and movie S4), suggesting that the HB-EGF released from the normal HaCaT cells activated the neighbouring CD9-overexpressed cells through a paracrine effect. Given all the above findings, we propose that CD9-mediated signalling coordinates the polarization of leader cells through a paracrine effect of HB-EGF, facilitating robust electrotaxis in the mixed-cell monolayer.

## Discussion

**Figure 7:**
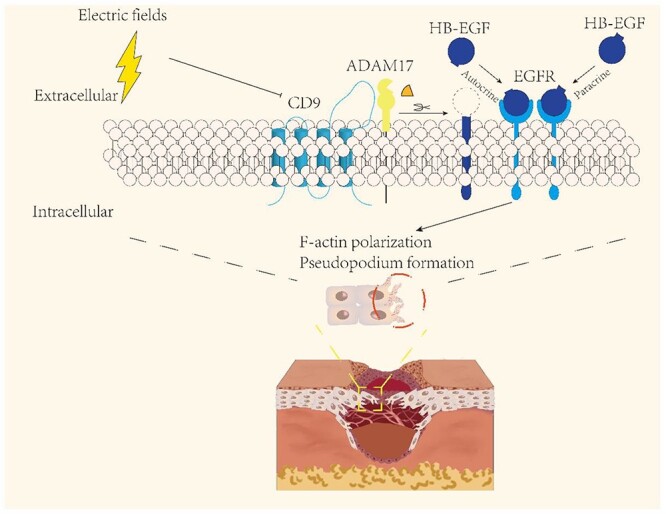
Schematic model depicts that CD9-mediated ADAM17/HB-EGF/EGFR signalling plays a key role in EF-guided collective migration by controlling the polarization of leader cells through autocrine and paracrine effects. *EFs* electrical fields, *CD9* tetraspanin-29, *ADAM17* a disintegrin and metalloprotease domain 17, *HB-EGF* heparin-binding EGF-like growth factor, *EGFR* epidermal growth factor receptor, *F-actin* fibrous actin

Acute and chronic skin wounds are a significant threat to public health. Aiming at restoring injured tissue, new methods based on regenerative medicine, tissue engineering, biomaterials and biological physics have attracted much attention during the past few decades [33–36]. In our previous study, we showed that the application of exogenous EFs significantly accelerated the migration of the neonatal epithelium and the re-epithelialization of wounds, providing *in vivo* evidence for the clinical application of bioelectric fields [37]. Nevertheless, the mechanism of EF-induced collective directional migration of epidermal cells remains unclear. Here, we identified that EFs activated the ADAM17/HB-EGF/EGFR axis by downregulating CD9 to induce and coordinate F-actin polarization and leader-cell induction, revealing a novel mechanism for the electrotactic migration of epidermal monolayers (Figure 7).

Although numerous studies have been conducted on the EF-guided migration of cells in isolation, they could not completely replicate wound healing *in vivo,* where healing occurs through epidermal collective migration [38]. Therefore, understanding the behaviour of the epidermal monolayer under EFs would be of great clinical significance. In fact, recent studies have described EF-induced collective migration in sheets of keratinocyte, corneal epithelial or mammary epithelial cells [9,39,40]. Compared with cells in isolation, the epithelial sheets exhibit greater sensitivity, more effective electrotaxis and directional persistence in responding to EFs [9]. In our study, we did find that EF at a strength as low as 50 mV/mm induced a robust electrotactic response in the epidermal monolayer, with a directedness of −0.70 (0.08) (Figure 1e).

Collective migration is a biological behaviour coordinated by two types of cells: leader cells and follower cells [41]. The formation of pseudopodia along the direction of migration is an important feature of leader cells by which they secure front–rear polarity in collective migration [42,43]. The formation of pseudopodia is known to be dependent on the redistribution of cellular F-actin [14,44]. In our study, F-actin was dynamically polarized along the leader edge of the cell monolayer under EFs; the polarization of F-actin towards the anode was increased 3.4-fold (Figure 2c). Depolymerization of F-actin by cytorelaxin B inhibited the formation of pseudopodia and the electrotactic migration of the epidermal monolayer, confirming F-actin polarization as the key to pseudopod formation and the initiation of the collective migration of cell sheets.

The induction of F-actin polarization in epidermal cells involves a series of events, including extracellular stimulation, membrane receptor activation and intracellular signalling [45,46]. Studies from Zhao *et al*. [47] and Fang *et al*. [48] have indicated a crucial role for HB-EGF/EGFR signalling in F-actin relocalization and polarization in a physiological EF. Our study further found that the release of HB-EGF was controlled by ADAM17 shedding, which indirectly induced EGFR activation and subsequent F-actin polarization in leader cells. More importantly, for the first time, we found that the activity of ADAM17 was negatively modulated by CD9, a highly conserved tetraspanin family protein that was shown to physically interact with ADAM17 in our previous study [20] and was found to be downregulated, particularly in leader cells, by EFs in this study. Overexpression of CD9 completely disrupted the polymerization of F-actin (Figure 5e). Therefore, F-actin polarization in leader cells is tightly controlled by CD9. In summary, CD9 acts as the key regulatory molecule upstream of the ADAM17/HB-EGF/EGFR axis in EF-guided collective migration of the epithelial monolayer. Future studies should determine how CD9 is downregulated by EFs and how it affects the activity of ADAM17 in leader cells of migrating epithelial monolayers under EFs.

The migration of epithelial monolayers is known to share signalling mechanisms with single cells that migrate in EFs. For example, Zhao *et al*. [49] have found that pharmacological blockade of PI3K signalling or knockout of the catalytic subunit of phosphoinositide 3-kinase (PI3K p110γ) abolishes the electrotaxis of both single cells and cell sheets. In our study, HB-EGF/EGFR signalling accounted for the EF-induced directional collective migration of the epithelial monolayer; it is also responsible for the electrotactic migration of epithelial cells in isolation [47,48]. However, there must be other, unique mechanisms that govern the coordinated movement of the cohesive cell monolayer, since epithelial monolayers exhibit more effective electrotaxis than isolated cells. Previous studies have identified that mechanical coupling between cells through E-cadherin is one of the key factors in the collective migration of epithelial sheets. When E-cadherin junctions are disrupted with antibodies, cell groups lose their coupling and their coordinated directional migration [9]. In our study, we revealed an important role for the paracrine effect of HB-EGF in the increased collective migration of the epithelial monolayer. We found that the abolished polarization of leader cells and directional migration of monolayers due to CD9 overexpression could be restored by the co-culture system; more importantly, this restoration was inhibited by HB-EGF antibody (Figure 6). These results provide evidence that HB-EGF released through CD9-mediated signalling can activate neighbouring cells through a paracrine effect, resulting in coordinated migration of monolayers under EFs.

## Conclusions

In conclusion, our findings suggest that EFs induce directional collective migration by epidermal monolayers through downregulation of CD9 in leader cells. This downregulation accounts for the polarization of leader cells by controlling F-actin redistribution through ADAM17/HB-EGF/EGFR signalling, and the released HB-EGF might also coordinate the polarization of neighbouring leader cells via its paracrine effects. Our study, therefore, not only sheds new light on the mechanisms of EF-guided collective migration of epidermal sheets but also may provide potential targets for the treatment of acute or chronic wounds.

## Abbreviations

ADAM17: A disintegrin and metalloprotease domain 17; Ad-CD9: CD9-overexpressing recombinant adenovirus; CD9: Tetraspanin-29; EGFR: Epidermal growth factor receptor; EF: Electric field; F-actin: Fibrous actin; HaCaT: Human immortalized keratinocytes; HB-EGF: heparin-binding epidermal growth factor-like growth factor; PBS: Phosphate buffer solution; p-EGFR: **P**hospho epidermal growth factor receptor; TAPI-2: TNF protease inhibitor 2; HCC Hepatocellular carcinoma; BCA Bicinchoninic acid assay; GFP Green fluorescent protein; SDS-PAGE Sodium dodecyl sulfate polyacrylamide gel electrophoresis; DAPI 4',6-diamidino-2-phenylindole; ANOVA Analysis of variance; CB Cytochalasin B; ELISA Enzyme-linked immunosorbnent assay; PI3K Phosphoinositide 3-kinase; TNF Tumor necrosis factor.

## Authors’ contributions

J.P.Z., J.L. and X.P.J. developed the initial concept and supervised the study; J.P.Z., J.L. and X.Q.L. designed the experiments; X.Q.L., J.L., J.R.Y., M.K., M.J., L.J.L., J.H.Z., Y.C., X.C., Z.Z. and C.W. performed the experiments and analyzed the data; J.L., J.P.Z. and X.P.J. contributed to the reagents, materials and analysis tools. X.Q.L., J.L. and J.P.Z. co-wrote the manuscript. XQ.L, JR.Y, M.K contributed equally to this work and share first authorship. 

## Conflicts of interest

None declared.

## Supplementary Material

movie_S1_tkad012

movie_S2_tkad012

movie_S3_tkad012

movie_S4_tkad012

Supplementary_File_for_Review_tkad012

## Founding

This study was supported by the National Natural Science Foundation of China (NSFC No. 82272285, NSFC No. 82002048 and NSFC No. 82072172), and the Scientific Research Project of Chongqing (CSTB2022BSXM-JCX0010). We are grateful to all the individuals who participated in this study. We would also like to thank the members of the Plastic Surgery, State Key Laboratory of Trauma, Burns and Combined Injury, Southwest Hospital for their helpful discussions.

## References

[1] Shaw TJ, Martin P. Wound repair at a glance. J Cell Sci. 2009;122:3209–13. 19726630 10.1242/jcs.031187 PMC2736861

[2] Xiao T, Yan Z, Xiao S, Xia Y. Proinflammatory cytokines regulate epidermal stem cells in wound epithelialization. Stem Cell Res Ther. 2020;11:232. 32527289 10.1186/s13287-020-01755-y PMC7291661

[3] Kruse CR, Singh M, Targosinski S, Sinha I, Sorensen JA, Eriksson E, et al. The effect of pH on cell viability, cell migration, cell proliferation, wound closure, and wound reepithelialization: in vitro and in vivo study. Wound Repair Regen. 2017;25:260–9. 28370923 10.1111/wrr.12526

[4] Sun YS . Electrical stimulation for wound-healing: simulation on the effect of electrode configurations. Biomed Res Int. 2017;2017:5289041. 28497054 10.1155/2017/5289041 PMC5401728

[5] Yang J, Liu X, Wang W, Chen Y, Liu J, Zhang Z, et al. Bioelectric fields coordinate wound contraction and re-epithelialization process to accelerate wound healing via promoting myofibroblast transformation. Bioelectrochemistry. 2022;148:108247. 35994901 10.1016/j.bioelechem.2022.108247

[6] Zhao M . Electrical fields in wound healing-an overriding signal that directs cell migration. Semin Cell Dev Biol. 2009;20:674–82. 19146969 10.1016/j.semcdb.2008.12.009

[7] Hart FX, Laird M, Riding A, Pullar CE. Keratinocyte galvanotaxis in combined DC and AC electric fields supports an electromechanical transduction sensing mechanism. Bioelectromagnetics. 2013;34:85–94. 22907479 10.1002/bem.21748

[8] Zhao M, McCaig CD, Agius-Fernandez A, Forrester JV, Araki-Sasaki K. Human corneal epithelial cells reorient and migrate cathodally in a small applied electric field. Curr Eye Res. 1997;16:973–84. 9330848 10.1076/ceyr.16.10.973.9014

[9] Li L, Hartley R, Reiss B, Sun Y, Pu J, Wu D, et al. E-cadherin plays an essential role in collective directional migration of large epithelial sheets. Cell Mol Life Sci. 2012;69:2779–89. 22410739 10.1007/s00018-012-0951-3 PMC3459324

[10] Lalli ML, Asthagiri AR. Collective migration exhibits greater sensitivity but slower dynamics of alignment to applied electric fields. Cell Mol Bioeng. 2015;8:247–57. 26692908 10.1007/s12195-015-0383-x PMC4675494

[11] Debets VE, Janssen LMC, Storm C. Enhanced persistence and collective migration in cooperatively aligning cell clusters. Biophys J. 2021;120:1483–97. 33617837 10.1016/j.bpj.2021.02.014 PMC8105737

[12] Mayor R, Etienne-Manneville S. The front and rear of collective cell migration. Nat Rev Mol Cell Biol. 2016;17:97–109. 26726037 10.1038/nrm.2015.14

[13] Qin L, Yang D, Yi W, Cao H, Xiao G. Roles of leader and follower cells in collective cell migration. Mol Biol Cell. 2021;32:1267–72. 34184941 10.1091/mbc.E20-10-0681 PMC8351552

[14] Schaks M, Giannone G, Rottner K. Actin dynamics in cell migration. Essays Biochem. 2019;63:483–95. 31551324 10.1042/EBC20190015 PMC6823167

[15] Cook GA, Longhurst CM, Grgurevich S, Cholera S, Crossno JT, Jr, Jennings LK. Identification of CD9 extracellular domains important in regulation of CHO cell adhesion to fibronectin and fibronectin pericellular matrix assembly. Blood. 2002;100:4502–11. 12453879 10.1182/blood.V100.13.4502

[16] Chen S, Sun Y, Jin Z, Jing X. Functional and biochemical studies of CD9 in fibrosarcoma cell line. Mol Cell Biochem. 2011;350:89–99. 21161334 10.1007/s11010-010-0685-1

[17] Matthews AL, Noy PJ, Reyat JS, Tomlinson MG. Regulation of a disintegrin and metalloproteinase (ADAM) family sheddases ADAM10 and ADAM17: the emerging role of tetraspanins and rhomboids. Platelets. 2017;28:333–41. 27256961 10.1080/09537104.2016.1184751 PMC5490636

[18] Charrin S, Jouannet S, Boucheix C, Rubinstein E. Tetraspanins at a glance. J Cell Sci. 2014;127:3641–8. 25128561 10.1242/jcs.154906

[19] Hemler ME . Tetraspanin proteins promote multiple cancer stages. Nat Rev Cancer. 2014;14:49–60. 24505619 10.1038/nrc3640

[20] Liu J, Zhu G, Jia N, Wang W, Wang Y, Yin M, et al. CD9 regulates keratinocyte migration by negatively modulating the sheddase activity of ADAM17. Int J Biol Sci. 2019;15:493–506. 30745837 10.7150/ijbs.29404 PMC6367546

[21] Li Y, Ren Z, Wang Y, Dang YZ, Meng BX, Wang GD, et al. ADAM17 promotes cell migration and invasion through the integrin beta1 pathway in hepatocellular carcinoma. Exp Cell Res. 2018;370:373–82. 29966664 10.1016/j.yexcr.2018.06.039

[22] Soto-Gamez A, Chen D, Nabuurs AGE, Quax WJ, Demaria M, Boersma YL. A bispecific inhibitor of the EGFR/ADAM17 axis decreases cell proliferation and migration of EGFR-dependent cancer cells. Cancers (Basel). 2020;12:411. 32050662 10.3390/cancers12020411 PMC7072247

[23] Jia N, Liu J, Zhu G, Liang Y, Wang Y, Wang W, et al. Polarization of ADAM17-driven EGFR signalling in electric field-guided collective migration of epidermal sheets. J Cell Mol Med. 2020;24:14073–85. 33164313 10.1111/jcmm.16019 PMC7753989

[24] Combedazou A, Gayral S, Colombie N, Fougerat A, Laffargue M, Ramel D. Small GTPases orchestrate cell-cell communication during collective cell movement. Small GTPases. 2020;11:103–12. 28980871 10.1080/21541248.2017.1366965 PMC7053965

[25] Rappel WJ . Cell-cell communication during collective migration. Proc Natl Acad Sci U S A. 2016;113:1471–3. 26802125 10.1073/pnas.1524893113 PMC4760825

[26] Han Y, You X, Xing W, Zhang Z, Zou W. Paracrine and endocrine actions of bone-the functions of secretory proteins from osteoblasts, osteocytes, and osteoclasts. Bone Res. 2018;6:16. 29844945 10.1038/s41413-018-0019-6 PMC5967329

[27] Huising MO . Paracrine regulation of insulin secretion. Diabetologia. 2020;63:2057–63. 32894316 10.1007/s00125-020-05213-5 PMC7968070

[28] Kim S, Subramanian V, Abdel-Latif A, Lee S. Role of heparin-binding epidermal growth factor-like growth factor in oxidative stress-associated metabolic diseases. Metab Syndr Relat Disord. 2020;18:186–96. 32077785 10.1089/met.2019.0120 PMC7196370

[29] Lian C, Ruan L, Shang D, Wu Y, Lu Y, Lu P, et al. Heparin-binding epidermal growth factor-like growth factor as a potent target for breast cancer therapy. Cancer Biother Radiopharm. 2016;31:85–90. 27093342 10.1089/cbr.2015.1956

[30] Liu J, Guo X, Ren X, Tian H, Liang Y, Luo Z, et al. A novel FPCL model producing directional contraction through induction of fibroblast alignment by biphasic pulse direct current electric field. Exp Cell Res. 2018;371:426–34. 30201453 10.1016/j.yexcr.2018.09.003

[31] Zhang J, Calafiore M, Zeng Q, Zhang X, Huang Y, Li RA, et al. Electrically guiding migration of human induced pluripotent stem cells. Stem Cell Rev Rep. 2011;7:987–96. 21373881 10.1007/s12015-011-9247-5 PMC3226697

[32] Poukkula M, Hakala M, Pentinmikko N, Sweeney MO, Jansen S, Mattila J, et al. GMF promotes leading-edge dynamics and collective cell migration in vivo. Curr Biol. 2014;24:2533–40. 25308079 10.1016/j.cub.2014.08.066 PMC4252779

[33] Cai L, Xu D, Chen H, Wang L, Zhao Y. Designing bioactive micro−/nanomotors for engineered regeneration. Eng Regen. 2021;2:109–15.

[34] Luo Z, Che J, Sun L, Yang L, Zu Y, Wang H, et al. Microfluidic electrospray photo-crosslinkable κ-carrageenan microparticles for wound healing. Eng Regen. 2021;2:257–62.

[35] Yu Y, Wang Q, Wang C, Shang L. Living materials for regenerative medicine. Eng Regen. 2021;2:96–104.

[36] Luo R, Dai J, Zhang J, Li Z. Accelerated skin wound healing by electrical stimulation. Adv Healthc Mater. 2021;10:e2100557. 33945225 10.1002/adhm.202100557

[37] Liang Y, Tian H, Liu J, Lv Y, Wang Y, Zhang J, et al. Application of stable continuous external electric field promotes wound healing in pig wound model. Bioelectrochemistry. 2020;135:107578. 32534380 10.1016/j.bioelechem.2020.107578

[38] Grada A, Otero-Vinas M, Prieto-Castrillo F, Obagi Z, Falanga V. Research techniques made simple: analysis of collective cell migration using the wound healing assay. J Invest Dermatol. 2017;137:e11–6. 28110712 10.1016/j.jid.2016.11.020

[39] Zhu K, Hum NR, Reid B, Sun Q, Loots GG, Zhao M. Electric fields at breast cancer and cancer cell collective galvanotaxis. Sci Rep. 2020;10:8712. 32457381 10.1038/s41598-020-65566-0 PMC7250931

[40] Gao J, Raghunathan VK, Reid B, Wei D, Diaz RC, Russell P, et al. Biomimetic stochastic topography and electric fields synergistically enhance directional migration of corneal epithelial cells in a MMP-3-dependent manner. Acta Biomater. 2015;12:102–12. 25311684 10.1016/j.actbio.2014.10.007 PMC4798428

[41] Rorth P . Collective guidance of collective cell migration. Trends Cell Biol. 2007;17:575–9. 17996447 10.1016/j.tcb.2007.09.007

[42] Khalil AA, Friedl P. Determinants of leader cells in collective cell migration. Integr Biol (Camb). 2010;2:568–74. 20886167 10.1039/c0ib00052c

[43] Haeger A, Wolf K, Zegers MM, Friedl P. Collective cell migration: guidance principles and hierarchies. Trends Cell Biol. 2015;25:556–66. 26137890 10.1016/j.tcb.2015.06.003

[44] Freedman SL, Suarez C, Winkelman JD, Kovar DR, Voth GA, Dinner AR, et al. Mechanical and kinetic factors drive sorting of F-actin cross-linkers on bundles. Proc Natl Acad Sci U S A. 2019;116:16192–7. 31346091 10.1073/pnas.1820814116 PMC6697872

[45] Seetharaman S, Etienne-Manneville S. Cytoskeletal crosstalk in cell migration. Trends Cell Biol. 2020;30:720–35. 32674938 10.1016/j.tcb.2020.06.004

[46] Sackmann E . How actin/myosin crosstalks guide the adhesion, locomotion and polarization of cells. Biochim Biophys Acta. 2015;1853:3132–42. 26119326 10.1016/j.bbamcr.2015.06.012

[47] Zhao M, Pu J, Forrester JV, McCaig CD. Membrane lipids, EGF receptors, and intracellular signals colocalize and are polarized in epithelial cells moving directionally in a physiological electric field. FASEB J. 2002;16:857–9. 11967227 10.1096/fj.01-0811fje

[48] Fang KS, Ionides E, Oster G, Nuccitelli R, Isseroff RR. Epidermal growth factor receptor relocalization and kinase activity are necessary for directional migration of keratinocytes in DC electric fields. J Cell Sci. 1999;112:1967–78. 10341215 10.1242/jcs.112.12.1967

[49] Zhao M, Song B, Pu J, Wada T, Reid B, Tai G, et al. Electrical signals control wound healing through phosphatidylinositol-3-OH kinase-gamma and PTEN. Nature. 2006;442:457–60. 16871217 10.1038/nature04925

