# Supplementary material for: CD9 negatively regulates collective electrotaxis of the epidermal monolayer by controlling and coordinating the polarization of leader cells
Source: Burns Trauma. 2023 Jul 24;11:tkad012. doi: 10.1093/burnst/tkad012 (PMC10365154; doi:10.1093/burnst/tkad012)
Supplement: Supplementary_File_for_Review_tkad012 [file supplementary_file_for_review_tkad012.docx]

**
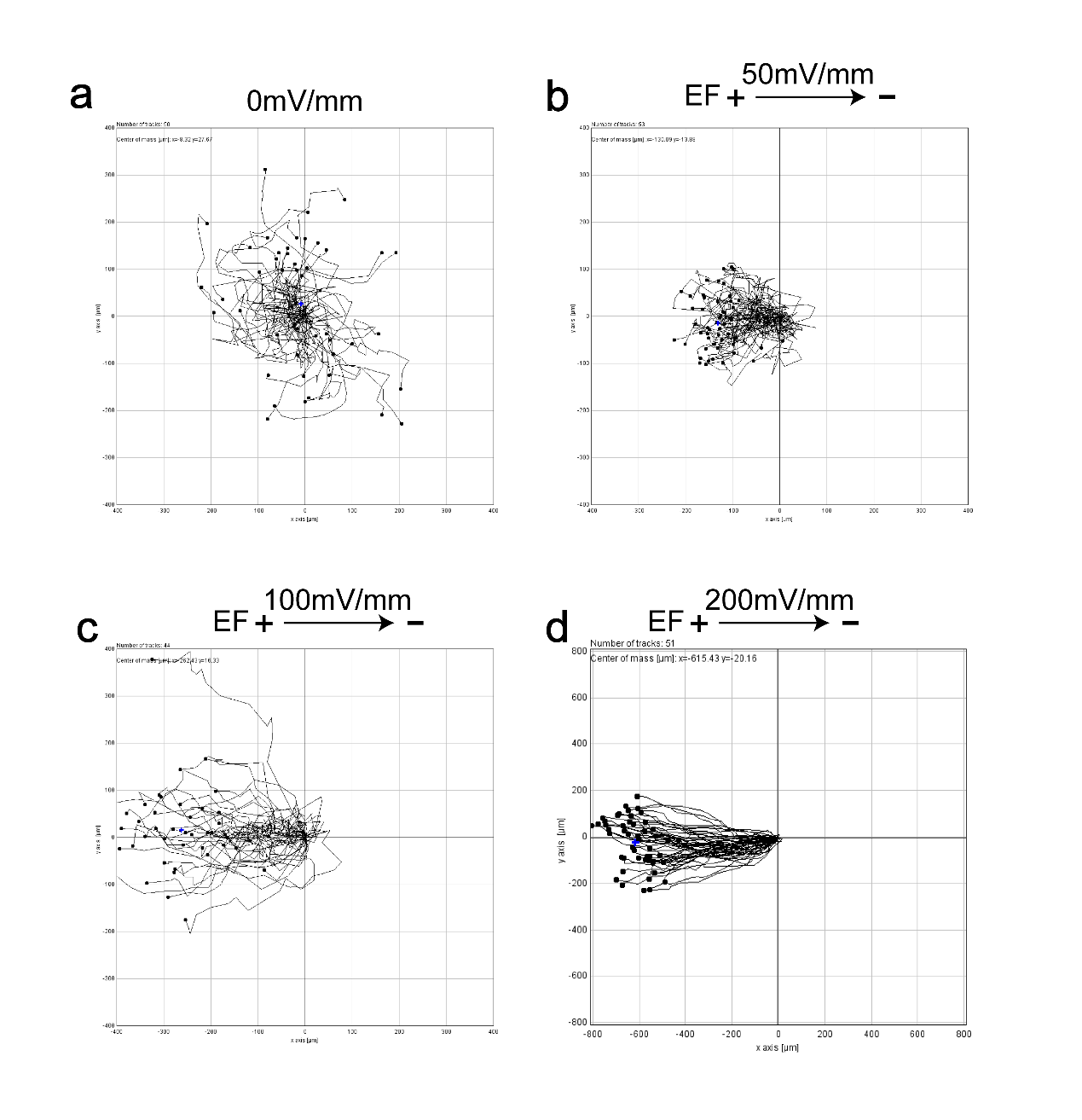
**

**Figure S1. EFs Induced directional collective migration of HaCaT monolayer to the Anode**. a，Migration trajectories of HaCaT monolayer in a EF of 0mV/mm(NO EF). b, Migration trajectories of HaCaT monolayer in a EF of 50mV/mm. c, Migration trajectories of HaCaT monolayer in a EF of 100mV/mm. d, Migration trajectories of HaCaT monolayer in a EF of 200mV/mm. EFs Electrical fields, HaCaT Human immortalized keratinocytes, EF Electrical field.


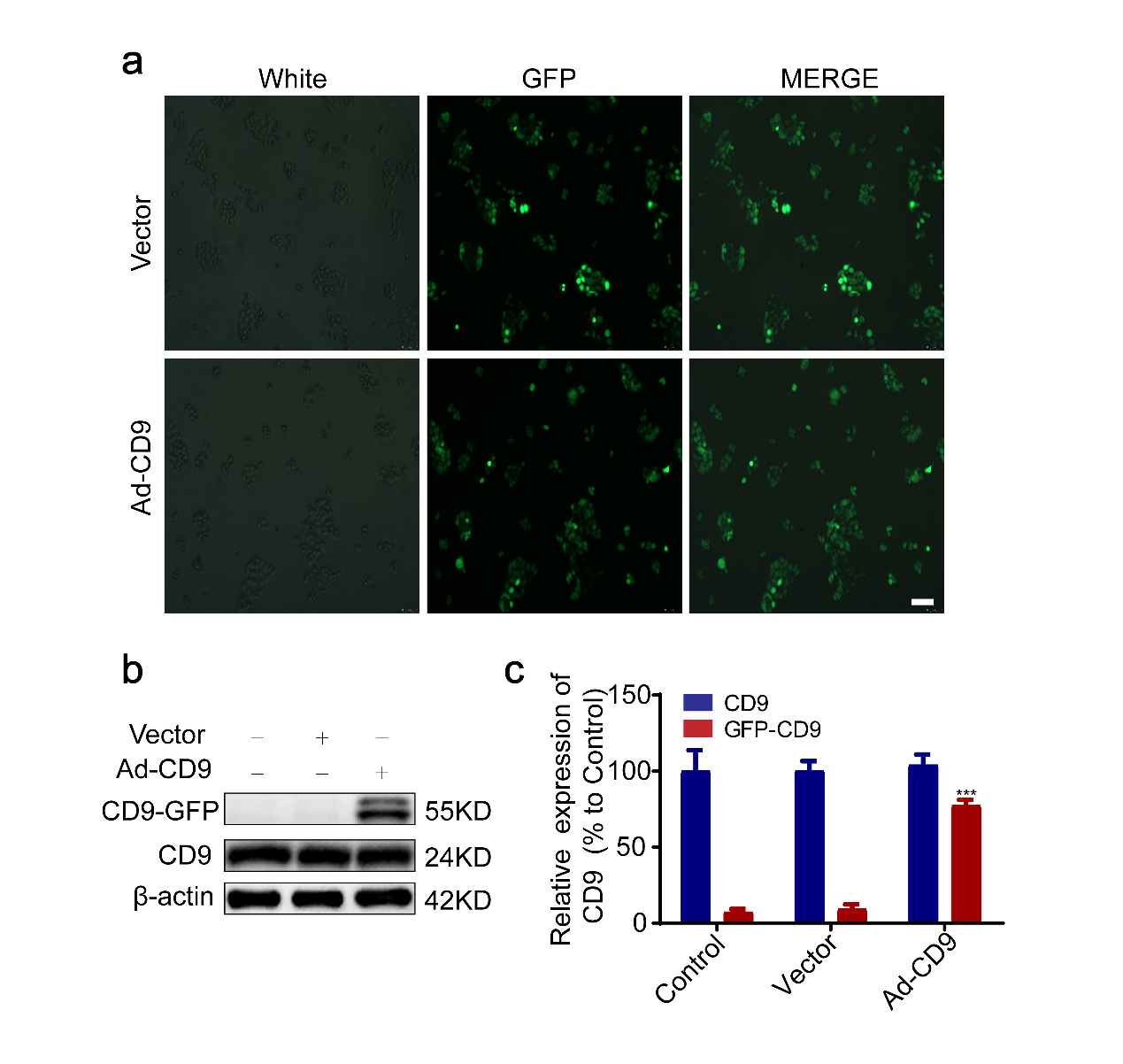


**Figure S2. HaCaT cells were infected with recombinant adenovirus vectors for overexpressing CD9 (Ad-CD9) and negative vectors (vector).** a, HaCaT cells were infected with mock vector (vector) or Ad-CD9 for 48h and then observed under a fluorescence microscope to determine the infection efficiency by visualizing expression of the gene for GFP. Bar=200μm. b, The expression of CD9 and CD9-GFP were determined by western blot. c, The results were quantified by relative intensity. ****P* <.001, vs. the Vector group. CD9 Tetraspanin-29, HaCaT Human immortalized keratinocytes, Vector Negative vectors, Ad-CD9 Adenovirus vectors for overexpressing CD9, GFP Green fluorescent protein.


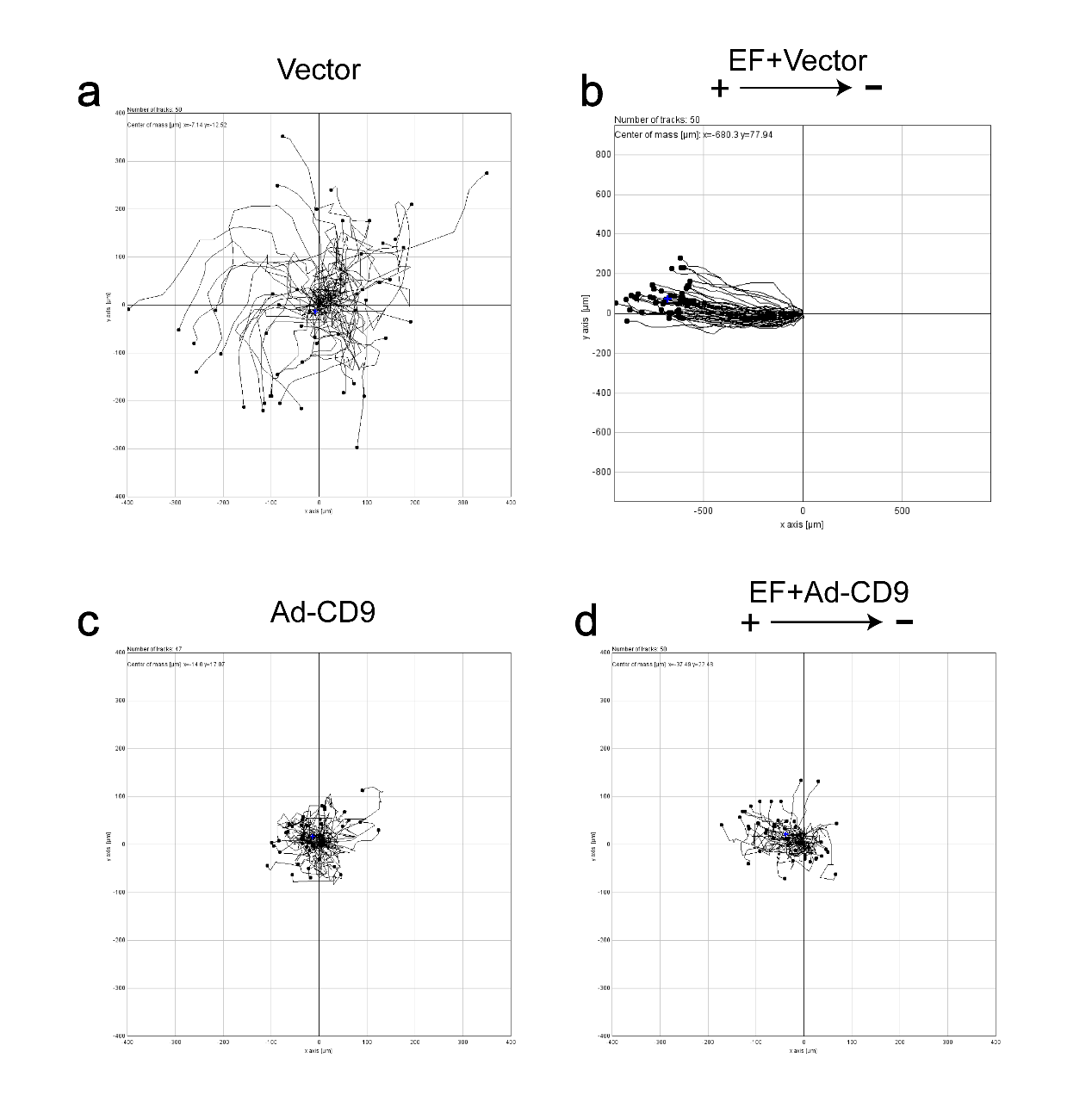


**Figure S3. Over-expression of CD9 inhibits the directional collective migration of HaCaT monolayer.** a,Migration trajectory of the HaCaT monolayer in Vector group. b, Migration trajectory of the HaCaT monolayer in EF+Vector group. c, Migration trajectory of the HaCaT monolayer in Ad-CD9 group. d, Migration trajectory of the HaCaT monolayer in EF+Ad-CD9 group. CD9 Tetraspanin-29, HaCaT Human immortalized keratinocytes, Vector Negative vectors, Ad-CD9 Adenovirus vectors for overexpressing CD9, EF Electrical field.


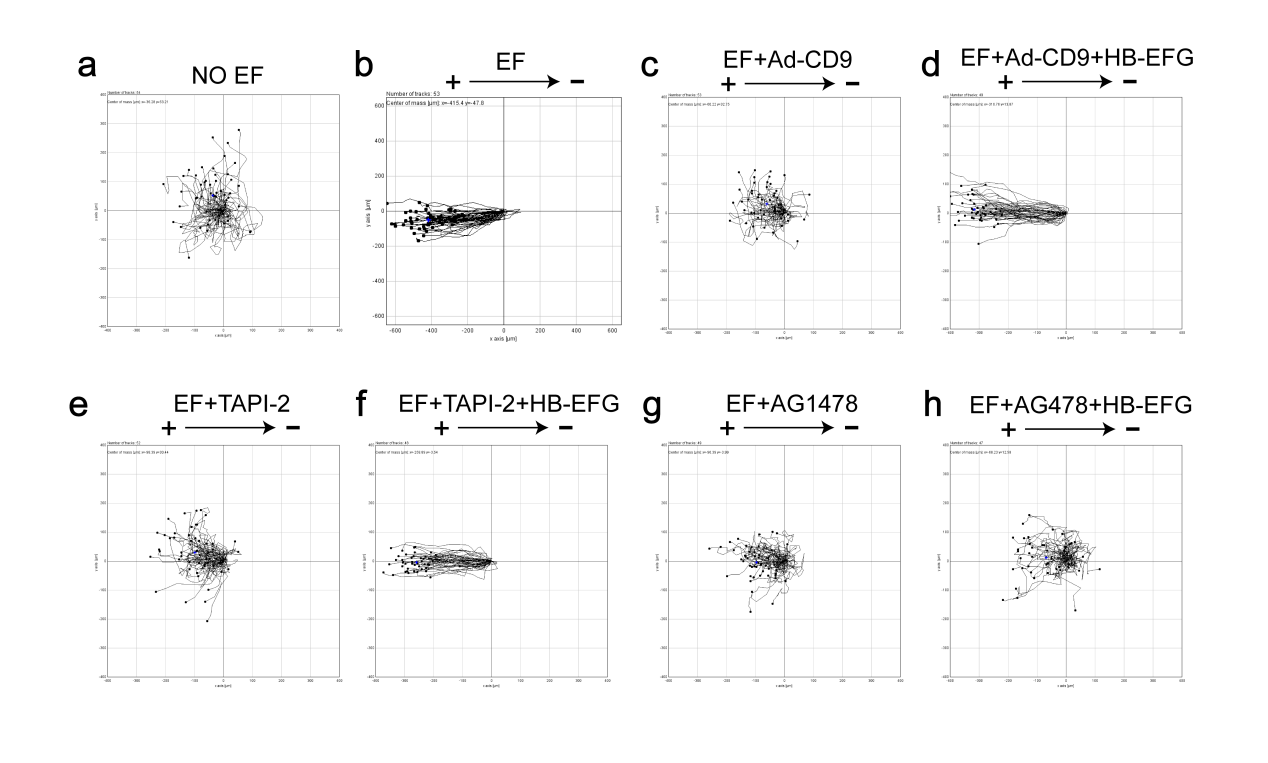


**Figure S4. Down-regulation of CD9 promoted collective electrotaxis depend on ADAM17/HB-EGF/EGFR axis under EFs.** a, Migration trajectory of the HaCaT monolayer in NO EF group. b, Migration trajectory of the HaCaT monolayer in EF group. c, Migration trajectory of the HaCaT monolayer in EF+Ad-CD9 group. d, Migration trajectory of the HaCaT monolayer in EF+Ad-CD9+HB-EGF group. e, Migration trajectory of the HaCaT monolayer in TAPI-2 group. f, Migration trajectory of the HaCaT monolayer in TAPI-2+HB-EGF group. g, Migration trajectory of the HaCaT monolayer in AG1478 group. h, Migration trajectory of the HaCaT monolayer in AG1478+HB-EGF group. CD9 Tetraspanin-29, ADAM17 A disintegrin and metalloprotease domain 17, HB-EGF Heparin-binding EGF-like growth factor, EGFR Epidermal growth factor receptor, p-EGFR Phospho epidermal growth factor receptor, TAPI-2 TNF protease inhibitor 2, AG1478 EGFR tyrosine kinase inhibitors, EFs Electrical fields, HaCaT Human immortalized keratinocytes, EF Electrical field, Ad-CD9 Adenovirus vectors for overexpressing CD9.


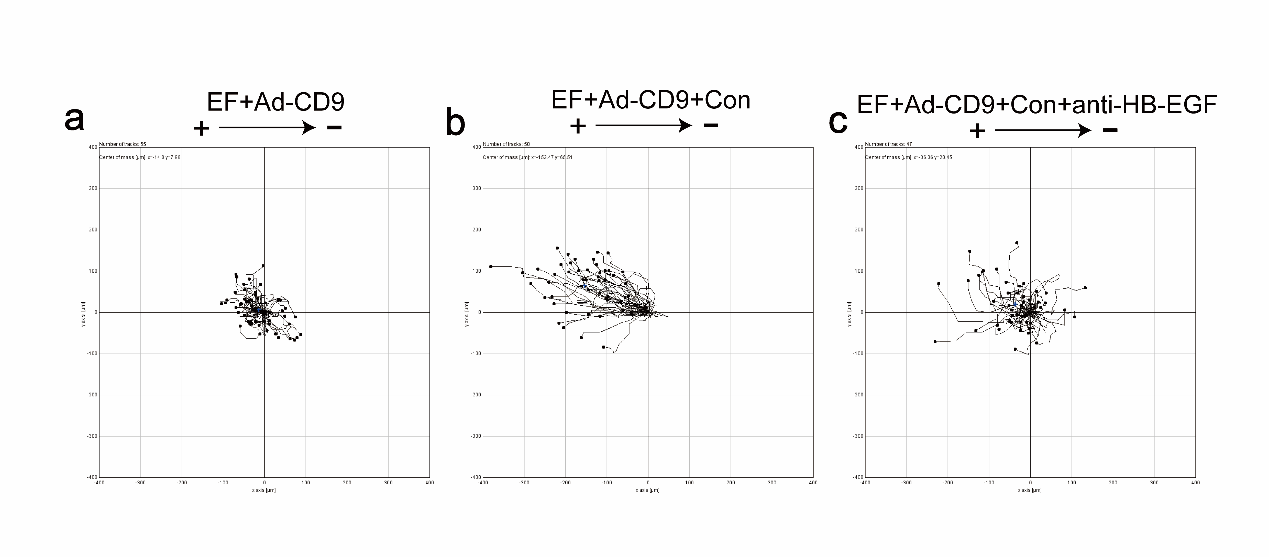


**Figure S5. Paracrine effect of HB-EGF released through CD9-mediated signaling coordinated the collective electrotaxis of HaCaT cells.** a, Migration trajectory of the HaCaT monolayer in EF+Ad-CD9 group. b, Migration trajectory of the HaCaT monolayer in EF+Ad-CD9+Con group. c, Migration trajectory of the HaCaT monolayer in EF+Ad-CD9+Con+anti-HB-EGF group. CD9 Tetraspanin-29, HB-EGF Heparin-binding EGF-like growth factor, anti-HB-EGF Heparin-binding EGF-like growth factor antibody, Con Control, HaCaT Human immortalized keratinocytes, EF Electrical field, Ad-CD9 Adenovirus vectors for overexpressing CD9.
